# The SFU Opinion and Comments Corpus: A Corpus for the Analysis of Online News Comments

**DOI:** 10.1007/s41701-019-00065-w

**Published:** 2019-11-02

**Authors:** Varada Kolhatkar, Hanhan Wu, Luca Cavasso, Emilie Francis, Kavan Shukla, Maite Taboada

**Affiliations:** 1 Department of Computer Science, University of British Columbia, Vancouver, Canada; 2 Discourse Processing Lab, Department of Linguistics, Simon Fraser University, Burnaby, Canada

**Keywords:** Constructiveness, Toxicity, Sentiment and opinion, Negation, Appraisal, News discourse, Online comments

## Abstract

We present the SFU Opinion and Comments Corpus (SOCC ), a collection of opinion articles and the comments posted in response to the articles. The articles include all the opinion pieces published in the Canadian newspaper *The Globe and Mail* in the 5-year period between 2012 and 2016, a total of 10,339 articles and 663,173 comments. SOCC is part of a project that investigates the linguistic characteristics of online comments. The corpus can be used to study a host of pragmatic phenomena. Among other aspects, researchers can explore: the connections between articles and comments; the connections of comments to each other; the types of topics discussed in comments; the nice (constructive) or mean (toxic) ways in which commenters respond to each other; how language is used to convey very specific types of evaluation; and how negation affects the interpretation of evaluative meaning in discourse. Our current focus is the study of constructiveness and evaluation in the comments. To that end, we have annotated a subset of the large corpus (1043 comments) with four layers of annotations: constructiveness, toxicity, negation and Appraisal (Martin and White, The language of evaluation, Palgrave, New York, 2005). This paper details our corpus, the data collection process, the characteristics of the corpus and describes the annotations. While our focus is comments posted in response to opinion news articles, the phenomena in this corpus are likely to be present in many commenting platforms: other news comments, comments and replies in fora such as Reddit, feedback on blogs, or YouTube comments.

## Introduction

Online commenting allows for direct communication among people and organizations from diverse socioeconomic classes and backgrounds on important issues. Popular news articles receive thousands of comments. These comments create a rich resource for linguists, as they provide examples of evaluative, abusive and argumentative language; sarcasm; dialogic structure; and occasionally well-informed constructive language. They contain information about people’s opinion or stance on important issues, policies, popular topics, and public figures. A number of interesting research questions about journalism, online language, and human conversation can be explored from such a resource.

Some of the research questions one could pose of online comments refer to their dialogic nature. Online comments can be characterized as polylogues (Marcoccia 2004), because they involve multiple levels of dialogue across participants. These questions revolve around the types of interactions and the nature of personal reference across participants, including attacks and abuse.

One could also examine the nature of rants, trolling and uncivil behaviour online. Although the comments in our corpus are moderated (see “Toxicity Annotations” section), disagreements and differences of opinion are still present. Studies of online rants (Lange 2014) show that rants are not always disruptive but help construct an online community. Similarly, Langlotz and Locher (2012) approached online disagreements as a way to explore the linguistic expression of emotional stance. Trolling has been explored from pragmatic (Jenks 2019) and corpus linguistic points of view (Hardaker 2015), but further work with different data such as what we present here is possible.

More generally, online comments are a fertile resource to explore many different linguistic characteristics and pragmatic structure. They are a source of irony, sarcasm and conversational humour, difficult to explore sometimes because of their decontextualized nature and the need to include multimodal material such as emoji. Some of these aspects have been studied for online language, for instance, irony in Twitter (Reyes et al. 2012), or negative sarcasm in language found online (Giora et al. 2014).

Specifically because of the type of annotations in the corpus, one could use the constructiveness annotations to study any possible differences in evaluative meaning between constructive and non-constructive comments. The negation annotations allow us to study how negation affects the interpretation of evaluative language in discourse. The corpus is also useful to research how to best organize comments to encourage constructive and civil conversations online, so that we can build better moderation systems.

In order to study some of these questions systematically and in particular to build moderation systems, we need a large, well-curated corpus of reader comments. Currently, only a few such corpora are available. One of them is the Yahoo News Annotated Comments Corpus (YNACC) (Napoles et al. 2017).^1^ This corpus contains 522,000 comments from 140,000 threads posted in response to Yahoo News articles. Among these, 9200 comments and 2400 threads were annotated at the comment level and the thread level. The comment-level annotations capture characteristics such as sentiment, persuasiveness or tone of each comment; whereas thread-level annotations label the quality of the overall thread such as whether the conversation is constructive and whether the conversation is positive/respectful or aggressive. The other prominent comments corpus is the SENSEI Social Media Annotated Corpus^2^ (Barker and Gaizauskas 2016). The goal of this work is to create summaries of reader comments, and accordingly, the authors created an annotated corpus of 1845 comments posted on 18 articles from the British newspaper *The Guardian*. The annotations were performed in four stages. First, each comment is labeled with a summary of the main points and the arguments or propositions expressed in the comment. The label is a short, free text annotation, capturing its essential content. Second, comments with similar or related labels are grouped together. Each group is assigned a label, which describes the common theme of the group, for example, in terms of topic, propositions, or contradicting viewpoints. Third, annotators write unconstrained and constrained summaries based on the group labels and their analysis of the groups. Finally, the annotators link back the sentences in their constrained summaries to the groups that supported that sentence.

The available corpora contain rich sources of information. We were interested, however, in the link between articles and comments, and in particular in how evaluative language varies between articles that contain opinion and their comments. The SFU Opinion and Comments Corpus contributes to these efforts by providing a large dataset with pairings of articles and comments. Our corpus not only contains comments, but also the articles from which the comments originated. Furthermore, the articles are all opinion articles, not hard news articles. This is important, because it allows for comparisons of evaluative language in both text types, opinion articles and reader comments. Opinion articles are generally subjective and evaluative, but their language tends to be more formal and argumentative. The comments are also subjective; they, however, tend to be more informal and personal in nature. The corpus is larger than any other currently available comments corpora, and has been collected with attention to preserving reply structures and other metadata. An example comment thread with replies is presented in (1), a set of comments replying to an article on a terrorist attack in Ottawa in 2014.^3^ The numbering system that starts with 6 points to this being the seventh top-level comment posted (the counter starts at 0). Comment 6_0 is a reply to that top-level comment, as is 6_1, whereas 6_1_0 is a reply to 6_1. The thread structure will be explained in more detail in the “Overview” section.

  6.Harper has some good ideas but one reason he does not get my vote is he is trying to turn the country into a police state.
    6_0. What good ideas? Tell me about them.
    6_1. Relax. He’ll never try what Saint Pierre Trudeau did. After two political kidnappings by Quebec separatists in 1970, the Trudeau government declared martial law in Quebec and Ottawa by bringing in the War Measures Act. Thousands of troops were put into the streets and hundreds of people were arrested without charges - some kept in jail for weeks.
      6_1_0. He can’t, that Act is no longer on the books.
      6_1_1. And the FLQ was crushed.
      6_1_2. You seem to have forgotten about the fact that 8 people were killed and over 90 bombinbings carried out before the War Measures Act was invoked.

In addition to the raw corpus, we also present annotations for four different phenomena: constructiveness, toxicity, negation and its scope, and Appraisal (Martin and White 2005), all defined later in the paper. We believe the corpus will be an invaluable resource for those interested in the language of evaluation, a number of linguistic and pragmatic phenomena, and how public opinion is expressed through comments.

In the next sections, we describe the raw and annotated portions of the corpus in detail, explaining the data collection and annotation methods, and the structure of the corpus. The corpus is publicly available; download links to the raw and annotated versions can be found on our GitHub page.^4^ We have also made separately available all the code and scripts used to find articles, scrape them and clean up the data.^5^

The data was collected under the fair dealing provision in Canada’s *Copyright Act.* The fair dealing provision permits the use of copyright protected works without permission in certain circumstances, which includes research and educational purposes.

## The SFU Opinion and Comments Corpus (SOCC )

In this section, we describe our raw corpus of opinion articles and their corresponding comments.

### Overview

The corpus contains all 10,339 opinion articles (editorials, columns, and op-eds) together with their 663,173 comments from 303,665 comment threads, from the main Canadian daily newspaper in English, *The Globe and Mail*, for a 5-year period (from January 2012 to December 2016). We organize our corpus into three sub-corpora: the articles corpus, the comments corpus, and the comment-threads corpus.

*The Articles Corpus* This corpus has 10,339 opinion articles, among which 7797 articles have at least one comment. We have included the remaining 2542 articles, which did not receive any comments, in the corpus because they can be useful in studying what kind of articles receive commenters’ attention.^6^ The articles were written by 1628 different authors, and cover a variety of topics from politics and social issues to policies and technology. The articles corpus has 6,666,012 words. The corpus is organized as a CSV file. For each article, we provide: date, author, title, URL, the number of comments, the number of top-level comments, and the article text of that article. Detailed information for each field is available on the project’s GitHub page.^7^

*The Comments Corpus* The second sub-corpus of SOCC is the comments corpus. This corpus contains the reader comments posted in response to the opinion articles from the articles corpus. Unfortunately, these comments are no longer visible on the *Globe and Mail* online interface, as the commenting platform has since changed and only comments posted after this change (around the beginning of 2018) are visible. This is unfortunate, but it also makes the corpus invaluable, as it captures a time period for which no comments are publicly available. The comments sub-corpus is organized as a CSV file containing individual comments and their metadata with minimal duplicate information. The corpus contains all unique comments after removing duplicates and comments with large overlap. The corpus is useful to study individual comments, i.e., without considering their location in the comment thread structure. The comments corpus has 37,609,691 words. In the comments corpus, we provide article identifier, thread information, date, the commenter username, comment text, and the popularity of the comment, based on the number of ‘likes’ the comment received.

*The Comment-Threads Corpus* The third sub-corpus of SOCC is the comment-threads corpus. This corpus contains all unique comment threads. The corpus can be used to study online conversations. The number of comments from this corpus is different from the comments corpus because we keep all comments in a conversation intact, including duplicates. This corpus is also organized as a CSV file, and the conversation structure is encoded in a field called *comment_counter*. The position of a comment in a comment thread is encoded with numbers separated by underscores, depending upon the level of the comment. For instance:

- First top-level comment: source1_article-id_0
- First child of the top-level comment: source1_article-id_0_0
- Second child of the top-level comment: source1_article-id_0_1
- Grandchildren: source1_article-id_0_0_0, source1_article-id_0_0_1

The comment-threads corpus contain 303,665 threads and 773,716 comments. On average, there were three comments per thread. Table 1 shows some summary statistics of SOCC.

**Table 1:** Statistics of the SFU Opinion and Comments Corpus

Item	Number
*Articles corpus*	
Number of articles	10,339
Number of words in articles	6,666,012
Number of unique article authors	1628
Number of articles with comments	7797
Average number of comments per article	85
Average number of threads per article	39
Average number of top-level comments per article	35
*Comments corpus*	
Number of comments	663,173
Number of words in comments	37,609,691
Number of unique commenters	34,472
Number of top-level comments	272,787
Average number of comments per commenter	19
*Comment-threads corpus*	
Number of threads	303,665
Number of comments	773,716
Average number of comments per thread	3

### Data Collection Process

We focused on opinion articles (editorials, columns and op-eds) and their comments. The reason for choosing opinion articles is that these articles tend to receive interesting comments, as the articles themselves are more subjective than hard news articles. We are interested in the difference in subjectivity and evaluative language between articles and comments. The reason for choosing *The Globe and Mail* is that it is the main nationally distributed newspaper in Canada with 6.5 million weekly readers in print and digital.^8^ Unfortunately, *The Globe and Mail* does not have an API, so we wrote a scraper to get opinion articles and their comments.^9^ Our data is made publicly available under the fair dealing provision in Canada’s *Copyright Act*, which permits the use of copyrighted protected works for research and education purposes.

**Fig. 1:**
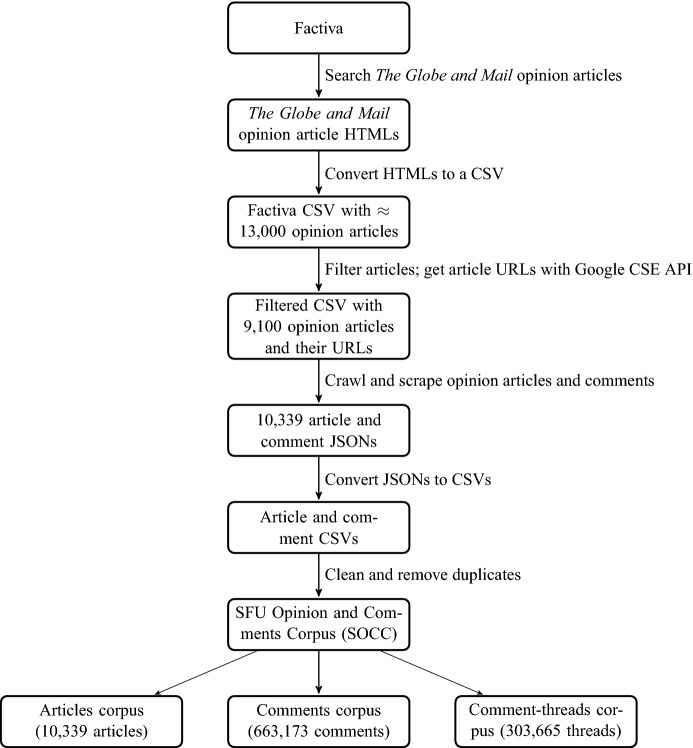
SOCC construction process

Figure 1 shows our corpus construction process and below we describe important steps in this process in detail.

*Collecting Only Opinion Articles* We were interested only in opinion articles and did not want to crawl and scrape all articles on the site. We also wanted to restrict our searches to a specific time period. This proved impossible using only the URLs on the site, as they did not have a structure showing section of the paper or date. As a result, to obtain a complete list of all opinion articles published in the relevant time period, we used Factiva,^10^ a business information and research tool that aggregates content from both licensed and free sources and provides organizations with search, alerting, dissemination, and other information management capabilities.

**Fig. 2:**
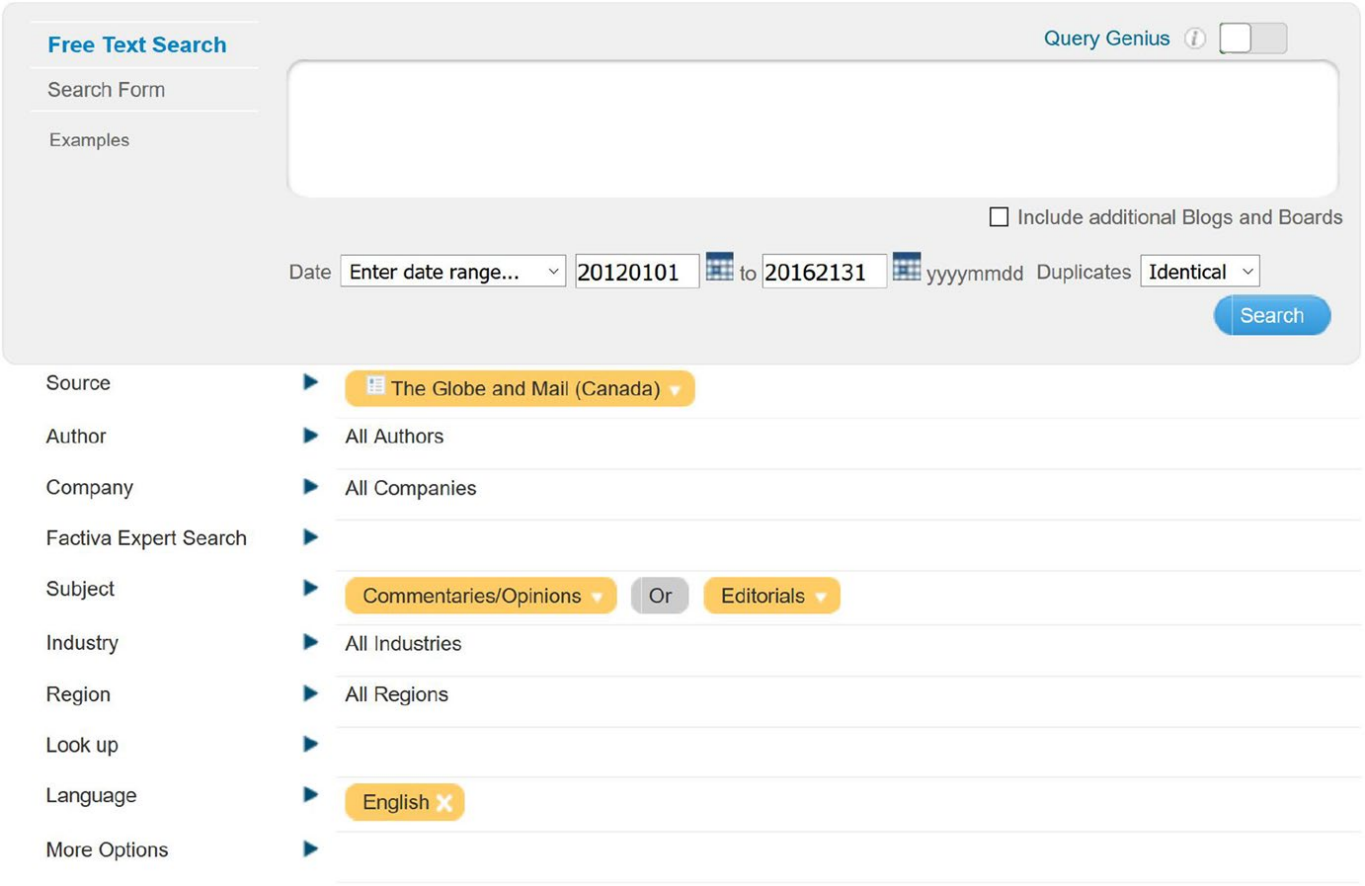
An example of the Factiva search interface and our search parameters

Factiva includes *The Globe and Mail *with same-day and archival coverage. Factiva does not offer an API but it has a search engine where certain parameters can be specified. The search interface is shown in Fig. 2. We searched Factiva for *Globe and Mail* opinion articles with the following search parameters.

- *Date* We selected the date range to be 20120101 (January 01, 2012) to 20161231 (December 31, 2016).
- *Duplicates* We selected *Identical* from the drop down menu, so that Factiva can remove duplicates.
- *SourceThe Globe and Mail (Canada)*.
- *SubjectEditorials* or *Commentaries/Opinions* under *Content Types* option.
- *LanguageEnglish*.

When searched with these parameters, Factiva returned $\approx$ 13,000 results. The interface shows 100 results per page, with various sorting and display options. We sorted the results by oldest first. For results display, we selected Full Article/Report plus Indexing option. We manually saved each result page as an HTML document. An example of a Factiva article with indexing is shown in “Appendix A”. Later we converted the saved HTML files into a comma-separated values (CSV) file, where each metadata field represents a column (e.g., the metadata field LP (lede paragraph)^11^ is a column in this CSV file). The CSV file contains all metadata from the Factiva data. Upon close inspection, among the over 13,000 articles returned, we discovered that many “columns” were not really opinion articles, but rather advice columns, listings for events, or articles in specific sections such as health.^12^ We identified the labels for those (they had the label ‘Column’ in the article type) and removed them from consideration, as they do not express opinion in the traditional sense (editorials or op-eds).

*Finding Opinion Article URLs from Opinion Articles in Factiva* Factiva contains article text and its metadata. However, it neither includes reader comments nor the article URL to scrape article comments. We found URLs by creating search queries from the Factiva CSV and then using these search queries with two different search engines. We created search queries using the first few sentences of the article text or the lede paragraph text, and then we searched for the appropriate URLs with the Google Custom Search Engine (CSE) API. We restricted the search parameters so that we only looked for the text on the *Globe and Mail* website. The CSE API places a restriction on the number of searches per day and thus this process of gathering URLs for opinion articles took a few weeks. Once we had article URLs, we added them to our Factiva CSV file. In a few cases (less than 200 cases), the Google CSE API did not return any results, and then we used the same search queries manually on the Bing search engine.^13^ In the end, we had 9100 opinion article URLs.

*Scraping the Globe and Mail*

We considered the set of URLs collected in the previous step as seed URLs for our crawler. The crawler crawled each of these seed URLs and looked for other opinion articles^14^ satisfying our year-range criteria. We started with 9100 distinct seed URLs, and ended up with 10,339 opinion articles between January 1, 2012 and December 31, 2016. We scraped these articles and their corresponding comments using scrapy, a Python library for extracting information from web sites.^15^ The scraped output is stored into JavaScript Object Notation (JSON) files.

Our comments are scraped from two different sources and with two different methods because, during our comment extraction process, *The Globe and Mail* changed their commenting structure. In particular, they added a *reactions* option for the commenters. Before this change, the commenters could only *like* or *dislike* a comment. With the new interface, commenters had a variety of options, such as *Like*, *Funny*, *Wow*, *Sad*, and *Disagree*. Because of this change, the comments posted before the date of the change (2016/11/28) disappeared from the website. We contacted *The Globe and Mail*, and they could not guarantee that the disappeared old comments would be recovered in the near future. Therefore, in our code, we have two separate methods to extract comments: the method to extract the old comments that were present before the comment structure change (not visible any more, but still accessible) and the method to extract new comments with commenter reactions. We refer to the first as source1 comments and the second as source2 comments. There was some overlap between the old and new comments, and thus we preprocessed the corpus to remove the duplicate comments. That said, if a thread from source1 is slightly different from a similar thread from source2 (only one comment is different), we kept both of these threads, considering them as two separate conversations.

*Organizing Data into CSV Files* To distribute the data, we organized the three sub-corpora into three comma-separated values (CSV) files.

1. **The articles corpus CSV (gnm_articles.csv)**
  This CSV contains opinion articles from *The Globe and Mail*, with the following fields.
  **article_id:** A unique article identifier, which is also used in the comments CSV.
  **title:** The headline of the article.
  **article_url:***The Globe and Mail* URL for the article.
  **author:** The author of the opinion article.
  **published_date:** The date of publication of the article.
  **ntop_level_comments:** The number of top-level comments for this article.
  **ncomments:** The number of all comments for this article.
  **article_text:** The article text with preserved paragraph structure.
2. **The comments corpus CSV (gnm_comments.csv)**
  A CSV containing individual comments and their metadata with minimal duplicate information. This CSV can be used to study individual comments in isolation, i.e., without considering their location in the comment thread structure.
  Below we list the most relevant columns of this CSV. Our GitHub project page contains an exhaustive list of all columns.
  **article_id:** The article identifier, also used in the article CSV.
  **comment_counter:** The comment counter, which is a unique comment identifier and encodes the location of the comment in the associated comment thread.
  **comment_text:** The comment text. We carried out minimal preprocessing on this text, where we deleted HTML characters (such as<p>) and added missing spaces after punctuation.
  **comment_author:** The author of the comment.
  **time_posted:** The time when the comment was posted.
3. **The comment-threads corpus CSV (gnm_comment_threads.csv)**
  A CSV containing comment threads and their metadata. In this CSV, we retain all comments in threads, which means there may be duplicate comments. The columns of this CSV are the same as the comments corpus CSV.

## Constructiveness and Toxicity Annotations in SOCC

There is growing interest in automatically organizing reader comments in a practical way (Llewellyn et al. 2014; Diakopoulos 2015; Napoles et al. 2017). One useful way to organize comments is based on their *constructiveness*, i.e., by identifying which comments provide insight and encourage a healthy discussion. For instance, *The New York Times* manually selects and highlights comments representing a range of diverse views, referred to as *NYT Picks*. The primary challenge in developing a computational system for automatically organizing comments in a sensible way is the lack of systematically annotated training data.

We annotate a sample of our SOCC corpus for constructiveness and toxicity level of individual comments. The goal of this annotation is twofold: first, to examine to what extent people agree on these notions and second, to examine the relationship between toxicity and constructiveness. The annotations are also useful to develop methods to detect constructiveness using computational linguistics techniques. Such methods are useful in developing moderation platforms. While most moderation systems are deployed to filter out inappropriate, abusive and toxic comments, we believe the quality of online conversations can be improved by also promoting and highlighting constructive comments. In related work (Kolhatkar and Taboada 2017a, b), we have used these annotations to develop and test a moderation platform.^16^

### Definitions

Rather than providing dictionary definitions or relying on our intuitions, we decided to post an online survey, asking people what they thought a constructive comment was. This is a form of crowdsourcing a definition. We are interested in crowd definitions, because presumably it is that population that posts comments on news sites. We posted a survey through SurveyMonkey,^17^ requesting 100 answers to the question ‘What does *constructive* mean in the context of news comments?’. Representative samples of the answers are in Table 2.

**Table 2:** Sample answers to the question ‘What does *constructive* mean?’

Constructive	Non-constructive
Provides evidence-based information	Opinions without support
Offers an alternative viewpoint	Merely assigns blame
Builds up and does not tear down	Dismisses the terms of debate
Asks an informed question	Emotional reactions
Provides well-researched answers	Excessively flattering
Adds new information or provides a new perspective	Personal or derogatory
Is specific and references facts	Irrelevant or too general

Previous papers that have tackled the issue of constructiveness in online comments and discussions offer different definitions. Niculae and Danescu-Niculescu-Mizil (2016) define a constructive online discussion as one where the team involved in the discussion improves the potential of the individuals. That is, the individuals are better off (in a game) when their scores are higher than those they started out with. The definition of Napoles et al. (2017) is characterized as more traditional: comments that intend to be useful or helpful. They define constructiveness of online discussion in terms of ERICs—Engaging, Respectful, and/or Informative Conversations. In their annotation experiment, those were positively correlated with informative and persuasive comments, and negatively correlated with negative and mean comments. In our annotation experiment, we used the following definition of constructiveness which was inspired by our survey answers: *Constructive comments intend to create a civil dialogue through remarks that are relevant to the article and not intended to merely provoke an emotional response; they are typically targeted to specific points and supported by appropriate evidence.*

We propose the label *toxicity* for a range of phenomena, including verbal abuse, offensive comments and hate speech. A *toxic* comment is one that is likely to offend or cause distress. Previous definitions have included personal attacks (Wulczyn et al. 2017), abuse (Nobata et al. 2016), harassment (Bretschneider et al. 2014), threats (Spitzberg and Gawron 2016), use of profane, obscene or derogatory language (Sood et al. 2012; Wang et al. 2014; Davidson et al. 2017), inflammatory language (Wiebe et al. 2001), hate speech (Warner and Hirschberg 2012; Djuric et al. 2015; Waseem and Hovy 2016), or apply the more general term cyberbullying, which refers not only to the language used, but also to other disturbing online behaviour such as repeated messages or threat of public exposure (Reynolds et al. 2011; Pieschl et al. 2015). For our annotations, we define toxicity on a four-point-scale (Very toxic, Toxic, Mildly toxic, Not toxic), where each point on the scale is defined in terms of the following characteristics. The definition for *Very toxic* included comments which use harsh, offensive or abusive language; comments which include personal attacks or insults; or which are derogatory or demeaning. *Toxic* comments were sarcastic, containing ridicule or aggressive disagreement. *Mildly toxic* comments were described as those which may be considered toxic only by some people, or which express anger and frustration. The following subsections detail the constructiveness and toxicity annotations. Full details, including instructions given to crowdworkers, are available from the corpus GitHub page.

### Constructiveness Annotations

We asked crowdworkers to annotate 1121 comments from SOCC for constructiveness and toxicity. Comments were selected from ten articles on various topics (the federal budget, a new national daycare plan, the Apple Watch, the results of the 2016 US Presidential election).^18^ We selected about 100 top-level comments for each article.

*Interface and Settings* We used CrowdFlower as our crowdsourcing interface, which has since been renamed Figure Eight.^19^ We asked annotators to read the article each comment refers to and to label the comment as constructive or not. For quality control, 100 comments were marked as gold: Annotators were allowed to continue with the annotation task only when their answers agreed with our answers to the gold questions. As we were interested in the verdict of fluent speakers of English, we limited the allowed demographic region to English-speaking countries (United Kingdom and Commonwealth countries where English is the majority language, although, naturally, the level of fluency varies across residents of these countries).

**Fig. 3:**
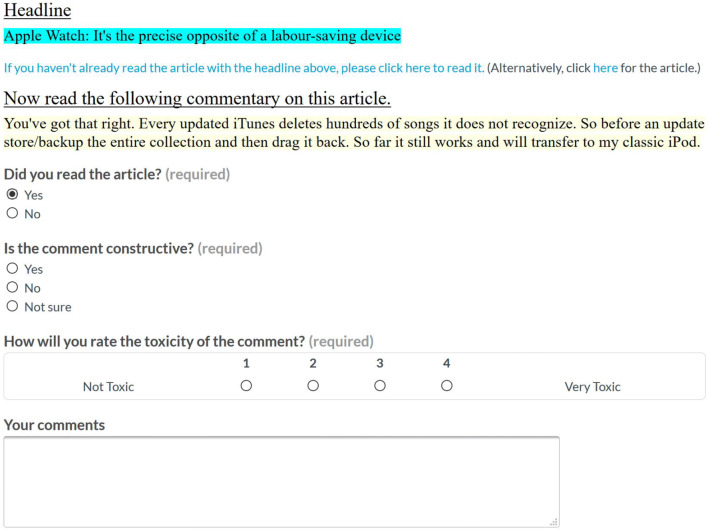
CrowdFlower interface to annotate constructiveness and toxicity

We asked for three judgments per instance (i.e., every comment was annotated by at least three people) and paid 5 cents per annotation. Annotators were shown the entire article, and then asked to annotate comments, one by one, and in random order. Comments are generally independent of each other, since they are top-level comments. In total, 72 different annotators were involved in the task, providing a total of 4238 judgements. The public release of the corpus provides all judgements for each comment, and also the majority vote. In other words, each comment has both a set of at least three judgements, but also a binary label with the majority vote (constructive or not constructive). Figure 3 shows our annotation interface.

*Agreement and Results* Percentage agreement for the constructiveness question on a random sample of 100 annotations was 87.88%, and Krippendorff’s alpha on the full dataset (three annotations per comment) was 0.49 (moderate agreement). These results suggest that constructiveness can be fairly reliably annotated. In our dataset, constructiveness is more or less equally distributed: Out of the 1121 comments, 603 comments (53.79%) were classified as constructive, 517 (46.12%) as non-constructive, and the annotators were not sure in only one case. Below we show examples of constructive and non-constructive comments from our corpus on an article on a newly-proposed national daycare plan.^20^ The comment shown in Example (2) is clearly a constructive comment.^21^ It is relevant to the article; it addresses a specific point (the cost of such a program); and it proposes an alternative solution, with a detailed description. The comment shown in Example (3), on the other hand, is not constructive. It dismisses the idea of a national daycare plan and criticizes the NDP’s approach without much explanation or evidence.^22^ Note that neither the constructive nor the non-constructive comment are particularly well written. For example, there are minor problems in both comments, such as missing punctuation or missing space after punctuation.

(2) While I support the notion of subsidized daycare, a national daycare program is an expensive boondoggle waiting to happen.A means tested subsidy paid directly to parents who use qualified facilities would create opportunities to increase available spaces. I would support a subsidy using the following guidelines:First, no family with income over $100,000 per year should require a subsidy. Subsidies could be scaled according to income levels from 25 to 75%. Second, people should expect to pay at least $10/day ($200/month) at any income level.Third, facilities that qualify for subsidies should be required to offer a minimum standard of service.
(3) Pay for your own kids’ babysitting.The last thing that any problem needs is an NDP style, big goverment, one-size fits all approach.

### Toxicity Annotations

In the context of filtering news comments, we are also interested in the relationship between constructiveness and toxicity. To better understand the nature of toxicity and its relationship with constructiveness, we included toxicity annotations in our CrowdFlower annotation. For the 1121 comments, we also asked annotators to identify toxicity. The question posed was: How toxic is the comment? As explained in “Definitions” section, we established four classes: *Very toxic*, *Toxic*, *Mildly toxic* and *Not toxic*. Figure 3 shows our annotation interface for annotating toxicity level. The annotation parameters are outlined in the Definitions section earlier (“Definitions” section) and described in full, with examples and screenshots, in the GitHub page for the corpus.

The percentage agreement for the toxicity question on a random sample of 100 annotations provided by CrowdFlower was 81.82% and Krippendorff’s alpha on the full dataset was 0.18, which indicates a very low agreement. We think agreement was low because the four classes were treated as mutually exclusive. It is quite likely that the class *Mildly toxic* for one person may be rather seen as *Toxic* by another annotator. Further analyses are needed to confirm this hypothesis.

The distribution of toxicity levels by constructiveness label is shown in Table 3. The most important result of this annotation experiment is that there were no significant differences in toxicity levels between constructive and non-constructive comments, i.e., constructive comments were as likely to be toxic (in its three categories) or non-toxic as non-constructive comments. The percentage distribution by toxicity level in Table 3 is quite similar across the two columns (constructive vs. non-constructive). For an illustration of the intersection between toxicity and constructiveness, consider Example (4) below. It was labelled as constructive by two out of three annotators and our expert, and toxic by all three and the expert, as it includes personal attacks on Trump and Clinton. It could be the case, in some situations, that a moderator may allow a somewhat toxic comment if it adds value to the conversation, i.e., if it is constructive.

(4) Please stop whining. Trump is a misogynist, racist buffoon and perhaps worse. Clinton is, to put it in the most polite terms possible, ethically challenged and craven in what she will tolerate in her lust for power. Neither of them is a stellar representative of their gender.Next time, put up a female candidate who outshines the male, not one who has sunk to his same level. Simple.

In summary, constructiveness and toxicity seem to be orthogonal categories. These results suggest that it is important to consider constructiveness of comments along with toxicity when filtering comments, as aggressive constructive debate might be a positive feature of online discussion. Given these results, the classification of constructiveness and toxicity should probably be treated as separate problems. The results, however, should be interpreted with caution, as our corpus has only moderated comments and the really toxic comments identified by the newspaper’s comment moderation system are not present in the corpus. We do not have much information about the type of moderation *The Globe and Mail* used at the time, but it seems that it was a combination of pre-moderation, with filters for abusive comments, probably based on keywords, and post-moderation, where readers flag comments that they believe are offensive. See Roberts (2019) and Gillespie (2018) for insightful discussions of different moderation approaches and the cost of human moderation.

**Table 3:** Percent distribution of constructive and toxic comments in CrowdFlower annotation

	Constructive ($n=603$) (%)	Non-constructive ($n=518$) (%)
Not toxic	82.09	78.57
Mildly toxic	16.08	15.44
Toxic	1.33	5.21
Very toxic	0.50	0.77
Total	100	100

### Expert Evaluation

To examine the quality of the crowd annotations we asked a professional moderator, with experience in creating and evaluating social media content, to evaluate the acceptability of the crowd’s answers. For that, we randomly selected 222 instances from the crowd-annotated data. We made sure to choose instances with medium confidence (0.6 $\le$ confidence < 0.9) and high confidence (0.9 $\le$ confidence $\le$ 1.0).^23^ We asked the expert whether they agreed with the crowd’s answer on constructiveness or not. We also asked them to rate the toxicity of the given comments on a scale of four toxicity levels. Given the high cost of an expert annotator, about 200 comments (roughly 20% of the data) was all that we could afford to annotate. We chose medium and high confidence instances because we wanted to determine whether the crowd’s opinion is comparable to that of an expert. In cases where confidence was low, we already know that the comments are difficult to assess. We add this layer of expert annotations in our constructiveness and toxicity corpus.

Overall, the expert agreed with the crowd 77.93% of the time on the constructiveness question. Among the 22.07% of cases where the expert did not agree with the crowd, 20.27% were marked as constructive by the crowd and the expert disagreed with these annotations. Here is the list of some of the prominent reasons why the expert thought the comments were non-constructive in these cases.

- Not enough content
- Not offering any real solutions or insights
- Assigns blame and is insulting and disrespectful
- Not relevant to the article
- Sarcastic and lacks evidence
- Intended to provoke an emotional response

Interestingly, there were four comments where the crowd thought the comments were not constructive, but the expert disagreed. An example is shown in (5). This comment was marked as non-constructive by our annotators, but the expert thought that the claims in the comment were supported by evidence.

(5) The numbers show that Democrats stayed at home and didn’t vote. She lost the support of her own party. In 2008 almost 80 million voted D and 50+ million R. That dwindled to about 65 million D and 50 million R in 2012. 2016 saw an even 50+ million to each party. Who’s fault is that?

To tackle the issues our expert pointed out, we are designing an annotation experiment where we ask specific questions on important aspects of constructiveness, such as whether the comment is relevant to the article, provides evidence or encourages dialogue, in addition to asking the binary question of whether the given comment is constructive or not.

### Duplicate Removal and Final Corpus

Our annotators found a few duplicate instances in our corpus. In particular, they noted some instances of equivalent comments, some with the text of the parent comment included in them and others without this text, as shown in Example (6).

(6)
  - Wow, Seiko’s cost that much? For half that you can buy a 75 year old Bulova, which will run another 75 years.
  - (In reply to:PRINCIPLE: The purpose of technology is to serve humankind, not the other way around. APPLICATION: Instead of buying a $500 Apple Watch, buy a $500 Seiko and enjoy a real watch.– Excimer) Wow, Seiko’s cost that much? For half that you can buy a 75 year old Bulova, which will run another 75 years.

We carefully curated the crowd annotated corpus and removed the instances containing the text of the parent comment. Note that our comments corpus and comment-threads corpus do not have such instances, as our preprocessing takes care of them. Since constructiveness and toxicity annotations were carried out before we cleaned SOCC , we carried out the duplicate removal process after the annotation process for this annotated corpus. The final annotated corpus, after duplicate removal, contains 1043 comments, which are organized into a CSV file. Below we describe the most relevant fields from this CSV. For information about the other fields and the corpus download link, please refer to our project’s GitHub page.^24^

- **article_id:** Article identifier for the article, which can be used to link the comment to the article corpus.
- **comment_counter:** Comment counter, which can be used to link the comment to the comments corpus or the comment-threads corpus.
- **comment_text:** Comment text shown to the annotators.
- **is_constructive:** Crowd annotation for constructiveness (yes/no/not sure).
- **toxicity_level:** Crowd annotation for toxicity level.
- **expert_is_constructive:** Expert annotation for constructiveness. Expert annotations are available for 200 of the comments.
- **expert_toxicity_level:** Expert annotation for toxicity level.
- **expert_comments:** Expert comments on crowd annotation.

The annotated corpus is useful to test classifiers for constructiveness and toxicity and to perform error analysis. We have used it for these purposes in related work (Kolhatkar and Taboada 2017a, b) and other researchers are also finding it useful (Risch and Krestel 2018; Lin et al. 2019).

## Negation Annotations on SOCC

Negation is a syntactic and pragmatic phenomenon that has both truth-conditional implications for the sentence, but also leads to pragmatic effects (Horn 1989; Potts 2011; Israel 2004). In this work, we are particularly interested in how to detect negation automatically, and how to interpret the effect it has on evaluative expressions.

The automatic identification and detection of negation has been a significant topic in biomedical text mining research (Aronow et al. 1999; Chapman et al. 2013; Mutalik et al. 2001). In the biomedical sphere, negation is often discussed in tandem with the concept of speculation (Vincze et al. 2008; Cruz Díaz et al. 2012). Negation and speculation are similar in that they affect the interpretation of the material in their scope. Consider sentences such as *It is not good* or *It may be good*, where the dictionary definition of *good* changes in the scope of negation (*not*) or speculation (*may*). Automatic methods for negation detection have also attracted much attention in the domain of sentiment analysis. Previous research has focused on classifying the scope of negation in relation to opinion in on-line reviews of products and films (Mittal et al. 2013; Dadvar et al. 2011; Councill et al. 2010). While earlier studies on negation have suggested some correlation between negation and negative affect (Potts 2011), it is not always the case that negation indicates negativity (Blanco and Moldovan 2014).

The primary goal of this research and annotation is to examine the relationship between negation, negativity, and Appraisal. It is clear that, in order to correctly interpret the sentiment expressed in online comments, an accurate interpretation of evaluative expressions in the scope of negation is necessary to fully capture the pragmatic effects of negation. We expand upon previous research in the field by considering the interaction between toxicity, constructiveness, and negation in online comments. For this purpose, we have created a unique and practical dataset containing comments annotated for negation. In doing so, we devised a formal strategy for annotating the focus of negation, a topic which has proven quite challenging in the past.

### Definitions

Before discussing the annotations, there are some essential terms which must be defined. The three core concepts customarily employed in negation analysis and annotation efforts are as follows.

*Keyword* A keyword is the element which triggers the negation. Keywords are a closed class of words, such as *no* or *not*, which project a scope and specify a focus. These negators are also referred to as negation cues or negative adverbs. We borrow the term ‘keyword’ from the literature on automatic negation detection (Vincze et al. 2008). A keyword tends to be at most one or two tokens. They are generally unambiguous and easily identified. Keywords include the negator *not*, whether by itself or attached to a verb (*the solution doesn’t lie in decolonizing*); other words such as *never*; and negative polarity items such as *nothing, nobody* or *nowhere* (Huddleston and Pullum 2002; Horn 1989). There are many potential negation cues (Morante and Sporleder 2012; Morante and Daelemans 2012), but we have focused on the most common ones.

*Scope* The scope of negation has been comprehensively researched in previous literature (Vincze et al. 2008; Jiménez-Zafra et al. 2018). It is defined as the part of the meaning that is being negated. In order to ensure that all elements which may plausibly fall within the scope are included, we adopted a maximal approach. Unlike the keyword and the focus, scope spans over the largest possible syntactic unit.

*Focus* Focus is the most elusive term to define. One definition is that the focus is the element which is intended to be false and is crucial for the interpretation of the negation (Vincze et al. 2008). Another definition of focus assumes that focus correlates with the answer to a wh-question (Rooth 1985). A third definition interprets focus based on a *question under discussion*, or QUD (Anand and Martell 2012). For this project, we adopt the definition in Blanco and Moldovan (2014). Focus is determined as the part of the meaning most explicitly negated. To keep focus concise, a minimal approach is adopted. Our motivation for a maximal approach in scope and a minimal one in focus relates to potential applications of the annotations. In applications such as sentiment analysis, where the goal is to accurately identify which word(s) in the sentence should have sentiment or valence revised. This is why focus has to be minimal, in order to zone in on the element most affected by the negation.

Example (7) shows a sample annotation. The keyword is *cannot*, with the negation attached to the modal verb. The scope is the VP after the keyword and the modal it is attached to, and the focus, i.e., the item most directly negated, is *believe*.

(7) I [cannot]$_{\textit{keyword}}$ [[believe]$_{\textit{focus}}$ that one of the suicide bombers was deported back to Belgium.]$_{\textit{scope}}$

### The Annotation Process

We annotated a total of 1121 comments for negation, using Webanno (de Castilho et al. 2016). After duplicate removal, the final annotations contain 1043 comments (see “Duplicate Removal and Final Corpus” section). Unlike the constructiveness and toxicity annotations, which were performed through crowdsourcing, negation and Appraisal were done in-house, by members of our research team and hired research assistants. The comments were annotated by up to two individuals, evaluated for agreement, then curated to arrive at the most precise annotation for each comment. Curation involved the more senior of the annotators making final decisions on which annotation to keep, in cases of disagreement. Annotations were done comment by comment, with each comment divided into sentences. We first identified the keyword, then the scope and finally the focus. Specific guidelines were developed to assist the annotators throughout the annotation process, and to ensure that annotations are standardized. These guidelines were developed based on previous annotation projects (Vincze et al. 2008; Jiménez-Zafra et al. 2018; Martín Valdivia et al. 2017) and improved upon to provide a thorough analysis of negation. Several versions of the guidelines were tested for accuracy of the annotations before deciding on the final version, which is available through the GitHub page for the corpus.^25^

There are four annotation labels associated with the negation project in WebAnno; these are *focus*, *scope*, *xscope*, and *neg* for keyword. Unlike previous annotation strategies which have included the keyword in the scope of the negation, the annotation system applied to this dataset uniquely excludes the keyword in the scope. This decision is motivated by the definition of scope adopted: the part of the meaning being negated. A keyword cannot negate itself, therefore it is more logical to conceive of the keyword as a flashlight which projects light, the scope, on other elements in the sentence.

In cases of elision or question and response, a special annotation label, *xscope*, was created to indicate the implied content of an inexplicit scope. Rather than considering instances of elision as negation without scope, it is assumed that the scope has simply been omitted and that, in most cases, it can be extrapolated from previous elements in the discourse. To indicate the context-derived scope of a negation involving omission, *xscope* is used, as shown in Example (8). Including *xcope* is useful in opinion mining, in case we want to identify an elliptical element negated in the current sentence or clause, but recoverable from the context. Aside from the two previously discussed deviations, the rest of the annotation approach follows prior conventions.

(8) He said he would [change the world]$_{\textit{xscope}}$, but he obviously [won’t]$_{\textit{keyword}}$

### Overview of Negation in the Corpus

After processing the corpus to remove duplicates, 1043 comments remained for preliminary analysis. Counting the spans for each label revealed that there were 1397 instances of *keyword*, 1349 instances of *scope*, 34 instances of *xscope*, and 1480 instances of *focus*. There are fewer instances of scope than of focus because, in some cases, both focus and scope are the same element, and in some cases the keyword has no scope (or scope that is not clearly recoverable from the linguistic co-text, in the form of xscope). In some cases, there are two foci per keyword. An example is provided in (8), where the two foci are *clear* and *rational*. Note that *is clear and rational* in the first clause is also the xscope for the keyword, as it appears in the previous clause.

(9) MHArding3’s point is [clear]$_{\textit{focus}}$ and [rational,]$_{\textit{focus}}$ yours is [not.]$_{\textit{keyword}}$

Most cases of negation contained an easily identifiable focus. In some cases, the main problem lay in determining the right contextual information. Although annotators read the article before annotating, they did not always have the full context. This issue is particularly exacerbated with sentences of considerable length. As a sentence containing negation became longer, it became more challenging to confidently ascertain the most appropriate focus. In Example (10), there is a particularly long clause which has multiple candidates for focus. Given the limited context, it is quite difficult to make a decision as any of the noun clauses within the scope are acceptable options. Ultimately, it was decided that the final element in the scope should bear the focus.

(10) So why [don’t]$_{\textit{keyword}}$ [moderate Muslims [head to places]$_{\textit{focus}}$ like Iraq and Syria and to other countries where Muslim extremists and terrorists exist to eradicate those Muslim radicals.]$_{\textit{scope}}$

The annotation process was not without challenges, mostly relating to interpreting ungrammatical and colloquial expressions. In some cases, ungrammatical sentences or multiple run-on sentences made it difficult to determine where a span should reasonably end. There were a great many comments within the corpus which included no instances of negation at all, whereas others included many. Of the 1043 curated comments, 401 did not contain any negation.

### Agreement

Two annotators performed the annotation. One was in charge of overseeing the process and training the research assistant, going through several iterations of annotation, refining the guidelines, and further annotation. The research assistant annotated the entire corpus. The senior annotator, one of the authors of this paper, then curated and made final decisions on any disagreements, so the entire corpus was annotated or curated by two different people. To calculate agreement, 50 comments from the beginning of the annotation process and 50 comments from the conclusion of the annotation process were compared.

Agreement was calculated using percentage agreement for nominal data, with annotations regarded as either agreeing or disagreeing. At the same time, we calculated Cohen’s kappa, to ensure agreement was not due to chance. We use Krippendorf’s alpha in the constructiveness and toxicity annotations (“Constructiveness Annotations” and “Toxicity Annotations” sections) because it can handle multiple annotators and different numbers of annotators per item (Artstein and Poesio 2008). In the negation and Appraisal annotations, on the other hand, there were always two annotators, and always the same annotators.

Agreement between the annotators was calculated first based on the span (whether the annotators agreed on the span of the item, e.g., the keyword), and then on the label (whether both annotators assigned the same label to the same span). By label we mean the tag used in the annotation, either keyword, scope, or focus. The annotations for scope include both scope and xscope, as xscope is considered a subtype of scope. For scope/xscope and focus, we considered agreement to occur when the two annotators agreed on span and label. There are three types of disagreement:

- *Label disagreement* The two annotators identified the same span, but gave it a different label (e.g., scope vs. xscope).
- *Selection disagreement* One annotator selected a span (and labelled it), but the other did not identify it as a span.
- *Span disagreement* Annotations partially overlap in span and have the same label. This usually means that the boundaries of the scope or the focus are slightly different.

The first 50 comments included a total of 51 instances of negation, that is, instances of a keyword. For those keywords, there were 43 cases of scope and xscope, and 49 cases of focus. Out of the annotations for keyword, a total of 49 were considered as agreeing (same span and same label). The two cases of disagreement were instances of selection disagreement.

In the annotations for scope, there were 38 instances of agreement, 2 label disagreements, 3 selection disagreements, and no span disagreement. The selection of focus showed more variability, a total of 49 elements were annotated for focus, but the label was agreed upon for only 23. There was 1 instance of label disagreement, 17 of selection disagreement, and 8 span disagreements. The percentage based results for the first 50 comments, as well as the average percentage of agreement, are shown in Table 4.

We calculated Cohen’s kappa on the keywords, that is, on the times that the annotators identified a keyword. We take the chance agreement to be 50%, based on the idea that, in a given unit (a sentence or a clause), the annotator will either find an instance of negation or not. The kappa value for the task of identifying a keyword was 0.92.

The results of this initial agreement study helped us refine our annotation process and gave us insight to carry out the rest of the annotation. We then performed a final agreement study.

The final 50 comments examined for negation included 82 keywords. There were 68 cases of scope and xscope, and 90 cases of focus. The keyword annotations exhibited 59 agreements. The 23 disagreements were all cases of selection disagreement. Cohen’s kappa for keyword identification was 0.39. For this second set, the annotations for scope showed 43 agreements, 1 label disagreement, 18 selection disagreements, and 6 span disagreements. The focus for the final 50 annotations, similar to the first 50, yielded a much lower percentage of agreement: There were 39 agreements, with no label disagreements, 47 selection disagreements, and 4 span disagreements.

**Table 4:** Percentage agreement for the first and last set of 50 comments

	Keyword (%)	Scope (%)	Focus (%)
Agreement first 50 comments	96	88	47
Agreement last 50 comments	70	63	43

From the results of the agreement calculations, the intuition that the keyword is the most easily identifiable feature is accurate. Whenever the annotators identify something as a keyword, there is obviously 100% agreement on its label. Instances of disagreement in the keyword are always attributed to an element being overlooked by either of the annotators (selection disagreement). The results for scope also show a relatively high percentage of agreement, suggesting that the scope is also fairly simple to determine. The results for focus, while noticeably lower than scope and keyword, are not surprising. Given the context-dependent nature of focus, disagreement is expected. It is sometimes difficult to identify the word or phrase most directly negated, particularly in instances where there are multiple candidates for focus. Recognizing the reliance on context, the agreement from these annotations is quite high. This suggests that the guidelines for annotation were beneficial for determining focus. One surprising effect was that agreement went down from the first 50 comments to the last 50, which were carried out over a period of three months. At the beginning of the annotation process, the lead annotator was working more closely with the other annotator to provide instruction as the novice became more comfortable with the procedure. This likely resulted in higher agreement between the two annotators as they would be in direct contact and influence each other’s opinions. Another reason for the loss in accuracy was perhaps haste towards the end of the annotation process. During curation, we observed that one of the annotators had overlooked keywords and their associated scope and focus. This was corrected in the curation process and in the final release of the corpus.

## Appraisal Annotations on SOCC

Following the framework of Martin and White (2005), we annotated Attitude,^26^ as well as Graduation of Attitude, using WebAnno (de Castilho et al. 2016). The Appraisal framework aims to capture the linguistic resources used to convey evaluation, and thus we thought it was the most appropriate framework to capture how evaluative language is expressed in the data, what types of evaluation are present, and the pragmatic meaning of evaluation in the corpus.

The motivation to choose Appraisal over other theories of evaluative meaning stems from our experience with Appraisal and the detailed set of labels it provides. The study of subjectivity, opinion and stance has yielded a rich literature, including many corpus studies of evaluation and stance in text. The work of Susan Hunston and Geoff Thompson has provided definitions and corpus-based analyses of evaluative meaning (e.g., Hunston and Thompson 2000; Alba-Juez and Thompson 2014; Hunston 2011). Corpus-based studies of stance in the framework of Biber and collagues (Biber and Finegan 1989; Conrad and Biber 2000) have shown interesting insights on evaluative language. We are interested in an analysis based on Appraisal because of the classification into different types of evaluation as stemming from personal feelings or evaluation of things and others. In particular the evaluation of other people (Judgement in the Appraisal framework) is useful in online comments, which are often aggressive in a personal way. There is a growing body of work on corpus approaches to Appraisal (see, among others, Lukin 2019; Breeze 2016; Hommerberg and Don 2015; Read and Carroll 2012; Bednarek 2009; Gales 2011; Love 2006; Aloy Mayo and Taboada 2017) and the methods for annotation and validation have been discussed in depth (Fuoli 2018; Fuoli and Hommerberg 2015; Taboada and Carretero 2012).

**Fig. 4:**
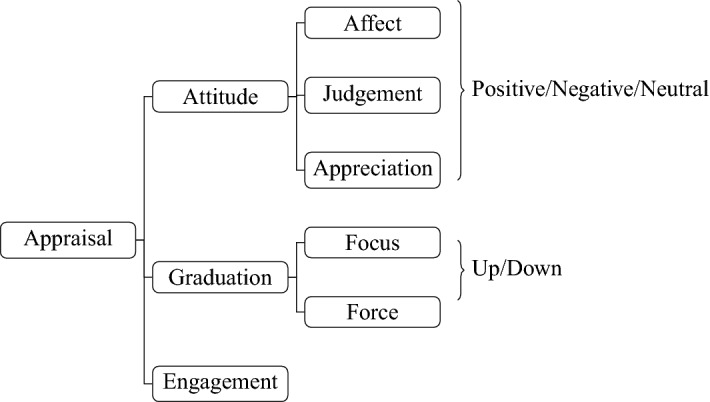
The appraisal system

In Appraisal, linguistic choices are characterized as systems of choices. The first of those choices is in the Attitude system, which in turn classifies evaluation as Affect (expressions of emotion by the speaker), Judgement (evaluation of other people’s abilities and ethics), or Appreciation (aesthetic evaluations of an object). Attitude can be intensified or downtoned through resources classified under Graduation. A third system, Engagement, characterizes resources to engage or disengage with the evaluation being expressed. In our annotations, we labelled linguistic expressions that convey Attitude and Graduation, but not Engagement, because the latter tends to be more difficult to identify and label, although negation is a resource used in Engagement, especially in the ‘deny’ aspect. Figure 4 contains a brief representation of the choices in Appraisal.

For our annotations, we found inspiration in existing work on manual annotation for Appraisal (Whitelaw et al. 2005; Read and Carroll 2012; Taboada et al. 2014), including the set of steps in Fuoli (2018) and Fuoli and Hommerberg (2015). The annotations consist of two layers: Attitude and Graduation. The Attitude layer labels spans by category (Affect, Appreciation or Judgement), as well as polarity (Positive, Negative or Neutral). The Graduation layer categorizes a span as either Force or Focus, with a polarity of up or down, i.e., an intensifying or downtoning effect. The pivot for all the annotations is the Attitude system. Once a span is identified as carrying Attitude (Affect, Judgement or Appreciation), then it is also annotated for polarity and, where applicable, Graduation. In other words, polarity and Graduation are labels that apply only when Attitude is present. Example annotations are given in (11). In the example, *pure politics* is an instance of negative Appreciation (the timing of the announcement was politically motivated). In addition, *pure* conveys Focus, which indicates that the word is most appropriate in this context. Since *politics* is presented as appropriate, this is ‘up’ Focus (otherwise known as ‘sharpen’ in Appraisal). Examples of ‘down’ (or ‘soften’) Focus are *more or less, kind of* of *somewhat*. The example also shows the interaction of negation and Appraisal, in *not good management*. In Example (12) we illustrate Force, and stacked annotations. For simplicity of presentation, we ignore the Appraisal in *quite normal* and *economic downturns* (which are both Appreciation, positive and negative, respectively). The interesting span here is *alleviate the problem to a degree*. We see an instance of negative Appreciation, *problem*, embedded in a larger span which conveys positive Appreciation, since the problem is alleviated. In addition, *to a degree* conveys Force, down, since it lessens the positive meaning of *alleviate*. The difference between Force and Focus is that Force applies to gradable words, whereas Focus is used with non-gradable expressions, such as *politics* in (11). Further details on how labels were used can be found in the Appraisal Guidelines document available from the download page for the corpus.

(11) The surplus and timing of the announcement is [pure politics,]$_{\textit{Appr,neg.Focus,up}}$ [not good management.]$_{\textit{Appr,neg}}$
(12) ...it is quite normal in times of economic downturns for governments to run deficits so as to [alleviate the [problem]$_{\textit{Appr,neg}}$ [to a degree]$_{\textit{Force,down}}$ ] $_{\textit{Appr,pos}}$

The Appraisal annotations provide a sophisticated level of analysis of evaluative language, based on a solid theory of how evaluation is conveyed through language. The annotations can prove useful in studies of the interplay of evaluative language, negation, and constructiveness and toxicity.

### The Annotation Process

Using WebAnno (de Castilho et al. 2016), a total of 1121 comments were annotated with the aforementioned labels according to the Annotation Guidelines (after duplicate removal, 1043 comments remain; see “Duplicate Removal and Final Corpus” section). As in the negation annotations, the comments were annotated by two individuals (one an author of this paper; the other a research assistant), evaluated for agreement, and curated to increase the accuracy of annotation. We began with guidelines previously used on a corpus of film reviews (Taboada et al. 2014) and the step-wise process in Fuoli (2018) and further developed them by iteratively testing and discussing those guidelines on our corpus. Once guidelines had been established, a research assistant annotated the original 1121 comments under the supervision of one of the researchers, who was responsible for curating the annotations. Curating involved examining each annotation instance, and ensuring that it conformed with the guidelines. When disagreement existed, the annotator and the curator discussed the source of disagreement, and the curator ultimately decided on the final label. This curation process was performed after the agreement measures were calculated.

Over the course of the annotation process, a few significant departures and clarifications needed to be made to the original guidelines. Originally, adjectives coordinated by a conjunction (13) were distinguished from those coordinated by a comma (14). This distinction was motivated initially by our previous work (Taboada et al. 2014), where we found that elements coordinated with *and* are part of the same evaluative unit and have the same label (both in terms of Attitude category and polarity). Elements coordinated with commas may indicate contrast rather than addition, and thus a different label for each element in the coordination. However, commenters did not seem to make a meaningful distinction between these two structures, which may be influenced by the difference in register between movie reviews and online comments on opinion articles.

(13) Hillary Clinton’s deeply flawed and frankly troubling history [...]
(14) Clinton is a power hungry, egotistical person [...]

Additionally, due to the content of the articles being commented on, many commenters expressed various attitudes towards people and situations. At times it can be ambiguous whether these attitudes represent Judgement or Appreciation, as they do in (15), where a mix of the two is present. There are two types of Attitude being expressed: one about the problems (in First Nations communities) and one about the asking (for resources or help), which is portrayed as *annoying and ridiculous*. These are Appreciation, as they refer to objects. The asking itself, however, is Judgement, as it is clearly the behaviour of the askers that is being judged. Likewise, the blaming is Judgement; *constantly blaming* is portrayed as Judgement. All of the instances in this example have negative polarity.

(15) Constantly [blaming]$_{\textit{Judg}}$ the rest of Canadians for the many aboriginal
  [problems]$_{\textit{Appr}}$ and [asking more and more]$_{\textit{Judg}}$ from other Canadians is [annoying and ridiculous.]$_{\textit{Appr}}$

Another issue in writing the guidelines was determining how long a span should be. In brief, we attempted to annotate spans that were as short as possible while containing all relevant words with attitudinal content. Many of the guidelines were designed to address cases where such a judgement is difficult to make, but as it is unfeasible to provide a guideline to cover every circumstance, there was inevitably some disagreement on the exact limits of a span.

### Preliminary Analysis

Over all 1043 comments, 6623 instances of Attitude and 771 instances of Graduation were found. Table 5 shows the number of spans that were annotated with each category and polarity of Attitude and Graduation.

Several very strong trends emerged from the data. Expression of Affect was quite rare in the corpus, comprising a mere 3.4% of the spans containing some kind of Attitude. As well, the Attitude expressed in these comments was overwhelmingly negative, as in nearly 75% of Attitude spans. Explicitly neutral positioning is rare, accounting for barely over one percent of the Attitude found in this corpus. It is, in fact, debatable whether Appraisal can be neutral, as an expression of evaluation tends to convey polarity. Cases of neutral Appraisal were most often negated positive or negative spans, e.g., *there is nothing racist about that*. Out of the 771 spans in which Graduation was found, Force used for up-scaling was prevalent. Out of all instances of Graduation, 85% were instances of Force, and 91% sharpened or scaled up the relevant Attitude.

The results contrast with our previous studies of Appraisal in movie reviews in English, German and Spanish (Taboada et al. 2014). There we also found that Affect was lower than Judgement and Appreciation, but not as low as in this corpus, varying between 20% in Spanish and 6% in German, with English in the middle at 15%. What was markedly different was that positive and negative spans were evenly distributed in movie reviews. Even in reviews that evaluated the movie negatively, giving it only one or two stars, there were many positive spans. By contrast, the comments in this corpus are predominantly negative, with almost 74% of the spans being negative.

**Table 5:** Instances of appraisal

	Frequency	Percentage
*Attitude type*		
Appreciation	3577	54.0
Judgement	2820	42.6
Affect	226	3.4
*Attitude polarity*		
Negative	4870	73.6
Positive	1679	25.3
Neutral	71	1.1
*Graduation type*		
Force	600	71.5
Focus	239	28.5
*Graduation direction*		
Up	637	75.9
Down	202	24.1

In future work, we intend to further analyze these patterns in terms of the genre of online comments and how negation affects Attitude. What these results show so far is that, surprisingly, Attitude is not expressed through personal feelings or emotions, which would receive the Affect label (*I hate X*), but rather as a third-person evaluation through Judgement or Appreciation, i.e., *X is a bad person; X is a bad idea*. The other interesting, but not surprising result is that Appraisal is predominantly negative. This confirms general online chatter about the nature of commenting, and is supported by numerous studies about negativity and toxicity online (Wulczyn et al. 2017; Cheng et al. 2017; Jeong 2018; Müller and Schwarz 2018).

### Inter-annotator Agreement

To determine agreement between the two annotators, two agreement studies were done, one once the research assistant indicated reasonable familiarity with the guidelines, and one approximately one and a half months later, at the end of the process. For each study, the research assistant and her supervisor annotated 50 comments in parallel.

Agreement was calculated separately for each layer (Attitude and Graduation) and each label within the annotation (category and polarity). One challenge to calculating agreement for this kind of annotation is that annotators sometimes disagree on the length of a span. As a result, we report Cohen’s Kappa for identical spans and overlapping spans, for both category (Attitude) and polarity labels. For identical spans, we counted the spans as agreeing if they were labeled the same, and disagreeing otherwise.

Agreement in overlapping spans was calculated as follows. All spans annotated with the same beginning, end and label were counted as agreeing. Spans which were labeled by one annotator but not the other were counted as disagreeing (selection disagreement, as defined for the negation annotations). When each annotator agreed on the start and end of a span but assigned it a different label, this was also counted as a disagreement (label disagreement). For spans which at least partially overlapped, agreement was decided on a case-by-case basis; if annotators were labeling similar phrases with similar labels, this was counted as an agreement, while using different labels or labeling different phrases were counted as disagreements (span disagreement for the latter case).

In addition to Cohen’s kappa, which adjusts for agreement based on chance, we calculated simple percentage agreement. As a raw percentage, agreement on Attitude category was at 81% in both the first and last 50 comments; agreement on its polarity was 89% for the first 50 and 87% for the last 50.

Agreement on Graduation was very low. As a raw percentage, it began at 41% in the first 50 annotations and ended at 45% for the final 50. This is because the research assistant did not have as much time to familiarize herself with the guidelines for Graduation as Attitude, and there are several ambiguities to be addressed for Graduation annotation. For example, in sentences such as (16) and (17), there is downscaling of a positive item (i.e., *substance, flexible,* and *able to handle change*). But in fact the negative Appreciation conveyed in those sentences is increased by this, as something which is less positive is effectively more negative. This was made clearer in the final guidelines document, where scaling was based on the Attitude expressed, so both of these would be classified ‘up’ for intensifying a negative.

(16) Apple stuff consist of 95% marketing nonsense and 5% substance
(17) [...] I suspect a narrow view is less flexible, less able to handle change.

*Agreement Study 1* The 50 comments in the first agreement study included 2688 words in 139 sentences. The results are summarized in Table 6, where we see moderate to good agreement numbers. Although no standard cut-offs for agreement exist, 0.67 is considered a point after which conclusions can be drawn from the data (Carletta 1996; Landis and Koch 1977), and 0.80 an acceptable level in most situations (Artstein and Poesio 2008). All of our numbers are within that range or higher.

**Table 6:** Cohen’s kappa results for the agreement study

	Identical spans only	Identical and overlapping spans
*First 50*		
Attitude type	0.69	0.72
Polarity	0.77	0.83
Average	0.73	0.77
*Last 50*		
Attitude type	0.71	0.71
Polarity	0.81	0.81
Average	0.76	0.76

There were many causes of disagreement in the annotations for the first 50 comments. Sometimes, the span of attitudinal content was unclear, as in (18), where the annotators disagreed as to whether *publicly call me a liar* or simply *call me a liar* should be annotated as negative Judgement. In this case, it was decided that *publicly* should be included in the span as publicly calling someone a liar is perhaps more face-threatening and thus more objectionable than doing so in private. These types of disagreements were addressed in the curation stage and settled for the final release of the corpus.

(18) I CANNOT allow you to publicly call me a liar!

Another issue causing disagreement was different interpretations of the appropriate label for an attitude-bearing span, as in (19) and (20). In both cases, the annotators disagreed as to whether the span in question was Appreciation or Judgement. Example (19) could be seen as Appreciation on the grounds that a continent with a dictator is institutionally bad, or as Judgement on the grounds that Merkel, as a supposed dictator, is reprehensible for her alleged dictatorship. Ultimately the interpretation as Judgement was deemed superior. Example (20) could be coded as Appreciation if it is interpreted more as a criticism of the interlocutor’s argument (along the lines of: *If the problem isn’t Islam, then why aren’t there radical Christian terrorists?*), or as Judgement due to its sarcastic tone and the implicit accusation of intellectual dishonesty or incompetence. This span was also deemed negative Judgement, especially because of its format as a rhetorical question, which can be seen elsewhere in the corpus as frequently accompanying and even conveying insults.

(19) The capital of Europe is Berlin and Merkel is the dictator.
(20) There are radical Christians causing world terror?

In addition to these disagreements, some spans were overlooked by one annotator or the other, likely due to the large volume of Attitude contained in each comment. Some disagreements are also attributable to errors based on unfamiliarity with the guidelines. There are also several errors based on combining spans conjoined by commas or the word *and,* as this guideline was not established until after agreement was calculated.

*Agreement Study 2* The 50 comments in the second agreement study included 3852 words in 207 sentences. The results of this study are also summarized in Table 6.

In the second set of 50 comments, many of the same issues as those in the first set reappeared. Another cause of disagreement is ambiguity in comments. For example, in (21), it is not obvious where the span(s) should begin and end, or whether the first part of the sentence is criticizing forced obsolescence (which would make it Appreciation) or Apple, the alleged corrupt purveyor of products which it supposedly forcibly obsolesces. In this case, we decided to annotate the entire span *the $13,000 gold model be obsolete when the next iteration is released in 3 months time* as negative Judgement, as an criticism towards the company. Similarly, in (22) it is not clear whether the commenter is sarcastically targeting the hypothetical iMplant and the institutional flaws of Apple, or the allegedly corrupt decision makers of Apple who supposedly want to invade people’s privacy by creating such a device. This example was also labelled as negative Judgement.

(21) Will the $13,000 gold model be obsolete when the next iteration is released in 3 months time [...]?
(22) Bahhh! I’ m waiting for the iMplant.$${}^{\mathrm{TM}}$$

Both the negation and Appraisal annotations were performed with the WebAnno interface. On our GitHub page, we provide download links for WebAnno files that can be explored with WebAnno, or as text files with annotations. The GitHub page also contains instructions for WebAnno installation.

## Conclusion

We have described The SFU Opinion and Comments Corpus (SOCC), a resource for exploring opinion news articles, online news comments and their relationship. A number of research questions related to journalism, online discourse, the dialogic structure of online comments, and the pragmatics of evaluative language can be explored from such a resource. We are particularly interested in using the annotations as a test dataset for a moderation system. Our current work examines whether we can promote and demote certain comments based on constructiveness and toxicity labels, and the annotations are useful for those purposes. We have developed a preliminary moderation system, a demo of which is available online: http://moderation.research.sfu.ca/. Other aspects that can be examined include the nature of evaluation, the role of negation in how evaluation is interpreted, and the relationship between articles and comments in terms of what articles and topics elicit more positive and negative comments.

Our corpus is composed of two kinds of sub-corpora: raw and annotated corpora. The raw corpus includes 10,339 opinion articles published in the Canadian newspaper *The Globe and Mail* in the 5-year period between 2012 and 2016, along with 663,173 comments and 303,665 comment threads in response to these articles. The annotated corpora comprises a subset containing 1043 comments from the raw corpus, enriched with constructiveness, toxicity, negation and Appraisal annotations.

While carrying out annotations for constructiveness and toxicity, we learned that constructiveness is an interplay between a variety of other phenomena of interest in pragmatics and computational linguistics, such as argumentation, relevance of the comment to the article and the tone of the comment. We believe that we may obtain better quality annotations if we ask specific questions leading to constructiveness (e.g., whether the comment is relevant to the article or whether the claims made in the article are supported by evidence), instead of asking a single binary question. In our current work we are pursuing this research direction.

With respect to the negation annotation, we developed extensive and detailed guidelines for the annotation of negative keywords, scope and focus. We used the guidelines to annotate the chosen subset of the comments corpus, producing a completely annotated corpus for negation, including its scope and focus. This corpus has been curated to provide the most accurate annotations according to the guidelines. We have also achieved reasonable results for agreement between annotators on these annotations.

With the Appraisal annotations we have shown that it is possible to achieve favourable rates of agreement using the guidelines, though agreement requires a high degree of familiarity with the guidelines and can still be hindered by ambiguity in comments. The corpus will be used to investigate the relationship between negation and Appraisal, but it has the potential for other avenues of research as well.

The corpus is freely available for non-commercial use. Full description of the corpus and structure: https://github.com/sfu-discourse-lab/SOCC and direct link to the data: https://researchdata.sfu.ca/islandora/object/islandora:9109.

## A Factiva Article with Metadata

See Table 7.

**Table 7:** An example Factiva article with metadata

SE	Editorial
HD	Not just words
WC	352 words
PD	30 December 2016
SN	The Globe and Mail
SC	GLOB
ED	Ontario
PG	A12
LA	English
CY	$\copyright$2016 The Globe and Mail Inc. All Rights Reserved
LP	There are 60 indigenous languages in Canada, more or less; the most spoken are Inuktitut and the related Cree and Ojibway. But while they are many, they risk disappearing. What is needed is a national effort to preserve them
TD	Senator Serge Joyal has been heroically trying to get a private member’s bill through the Senate to help revitalize indigenous languages. But that can’t possibly work, for the simple reason that a Senate bill can’t force the government to spend any money
	More promising is Prime Minister Justin Trudeau’s vow to introduce a bill to re-energize indigenous languages. He offered no details when he made the announcement, but it’s still progress
	For many, the urgent interest in this issue is about preserving dying languages, which are understood mostly by elderly people
	In an era of reconciliation with indigenous peoples, putting money into the preservation of native languages would be a concrete gesture that could produce equally concrete benefits
	Section 13 of the United Nations Declaration on the Rights of Indigenous Peoples, if adopted in a measured way, can be a helpful guide. It guarantees the right to the preservation of native languages and literature, and the right to retain native place names. It also guarantees the right to a trial in one’s native language, which would be too difficult to accommodate in all cases
	The teaching of indigenous languages should be a priority on reserves, especially the most remote ones. Young people there may be most in need of tools to help overcome their alienation
	But there can also be excitement and value in learning a language and culture that you don’t have any hereditary link to, something non-native Canadians might be interested in
	The University of Winnipeg’s compulsory policy that all its students take at least one course in an aboriginal subject goes too far
	But the intention behind that requirement is good
	These are some of the principles on which the government can form new policies on native languages—and not just for people with indigenous ancestry
NS	nedi : Editorials | ncat : Content Types
RE	cana : Canada | namz : North America
PUB	The Globe and Mail Inc.
AN	Document GLOB000020161230eccu0000c

## Footnotes

1 https://webscope.sandbox.yahoo.com/catalog.php?datatype=l&did=83.

2 http://www.sensei-conversation.eu/.

3 https://www.theglobeandmail.com/opinion/lone-wolves-police-state/article21354222/.

4 https://github.com/sfu-discourse-lab/SOCC.

5 https://github.com/sfu-discourse-lab/SFU_Comment_Extractor.

6 *The Globe and Mail,* at the time of data collection at least, allowed comments for all articles.

7 https://github.com/sfu-discourse-lab/SOCC.

8 https://www.theglobeandmail.com/report-on-business/globe-has-countrys-largest-weekly-readership-survey/article34119464.

9 We contacted *The Globe and Mail*, but were not able to obtain their help in the data collection process.

10 https://www.dowjones.com/products/factiva.

11 The lede is the opening paragraph of a news article, spelled this way in North American usage to distinguish it from *lead*, a strip of that metal used in early printing.

12 For example, https://www.theglobeandmail.com/life/health-and-fitness/ask-a-health-expert/which-granola-bars-are-the-healthiest-to-eat/article11703335.

13 https://www.bing.com.

14 We consider a scraped article as an opinion article if its URL starts with http://www.theglobeandmail.com/opinion.

15 https://scrapy.org/.

16 A demo of the moderation platform is available: http://moderation.research.sfu.ca/.

17 https://www.surveymonkey.com.

18 http://www.sfu.ca/discourse-lab/research/globe.html.

19 http://www.figure-eight.com.

20 https://www.theglobeandmail.com/opinion/daycare-picks-up-the-ndp/article21094039.

21 Examples are from our corpus, and are reproduced verbatim, including any non-standard spelling or grammar.

22 NDP = National Democratic Party, one of the main political parties in Canada.

23 In CrowdFlower terminology, each annotator has a trust level based on how they perform on the gold examples, and each answer has a *confidence*, which is a normalized score of the summation of the trusts associated with annotators.

24 https://github.com/sfu-discourse-lab/SOCC#constructiveness.

25 https://github.com/sfu-discourse-lab/SOCC/tree/master/guidelines.

26 We follow conventions in the Appraisal Framework and in Systemic Functional Linguistics to capitalize the first letter of the systems being described (Attitude, Appreciation, Graduation, etc.).
